# Probing the interaction interface of the GADD45β/MKK7 and MKK7/DTP3 complexes by chemical cross-linking mass spectrometry

**DOI:** 10.1016/j.ijbiomac.2018.03.090

**Published:** 2018-07-15

**Authors:** Camilla Rega, Rosita Russo, Annalia Focà, Annamaria Sandomenico, Emanuela Iaccarino, Domenico Raimondo, Edoardo Milanetti, Laura Tornatore, Guido Franzoso, Paolo Vincenzo Pedone, Menotti Ruvo, Angela Chambery

**Affiliations:** a Department of Environmental, Biological and Pharmaceutical Sciences and Technologies, University of Campania “Luigi Vanvitelli”, 81100 Caserta, Italy; b CNR-IBB, 80134 Napoli, Italy; c Department of Molecular Medicine, Sapienza University of Rome, 00161 Roma, Italy; d Department of Physics, Sapienza University of Rome, 00161 Rome, Italy; e Department of Medicine, Centre for Cell Signalling and Inflammation, Imperial College London, London W12 0NN, UK

**Keywords:** GADD45β, MKK7, Protein-protein interaction, Chemical cross-linking, Mass spectrometry

## Abstract

GADD45β is selectively and constitutively expressed in Multiple Myeloma cells, and this expression correlates with an unfavourable clinical outcome. GADD45β physically interacts with the JNK kinase, MKK7, inhibiting its activity to enable the survival of cancer cells. DTP3 is a small peptide inhibitor of the GADD45β/MKK7 complex and is able to restore MKK7/JNK activation, thereby promoting selective cell death of GADD45β-overexpressing cancer cells.

Enzymatic MS foot-printing and diazirine-based chemical cross-linking MS (CX-MS) strategies were applied to study the interactions between GADD45β and MKK7 kinase domain (MKK7_KD) and between DTP3 and MKK7_KD. Our data show that the binding between GADD45β and MKK7 largely occurs between GADD45β loop 2 (region 103–117) and the kinase enzymatic pocket. We also show that DTP3 interferes with this GADD45β/MKK7 interaction by contacting the MKK7 peptides, 113–136 and 259–274. Accordingly, an MKK7_KD Δ(101–136) variant lacking Trp135 did not produce a fluorescence quenching effect upon the binding of DTP3. The assessment of the interaction between GADD45β and MKK7 and the elucidation of the recognition surfaces between DTP3 and MKK7 significantly advance the understanding of the mechanism underlying the inhibition of the GADD45β/MKK7 interaction by DTP3 and pave the way to the design of small-molecule DTP3 analogues.

## 1 Introduction

Nuclear factor kappa-light-chain-enhancer of activated B cells (NF-κB) plays a crucial role in inflammation and cancer. Its activity is constitutively elevated in many types of human tumours, of either haematological or solid origin, and its inhibition can in principle counteract the oncogenic features of malignant cells [1]. In this respect, NF-κB-targeting strategies have promised much, but have so far failed to produce clinically useful therapeutics due to the emergence of dose-limiting toxicities. This reflects the widespread involvement of NF-κB in pleiotropic physiological functions, such as its functions in immunity, inflammation and tissue homeostasis. We have demonstrated [2] that an important mechanism underpinning the resistance to apoptosis of malignant cells from multiple myeloma (MM), a cancer of plasma cells, is the NF-κB-dependent up-regulation of the Growth arrest and DNA-damage-inducible GADD45-family member, GADD45β (Uniprot AC: O75293; 160 amino acids; Molecular mass 17,818 Da). Our study also showed that constitutive GADD45β expression is largely restricted to cancer cells, where it fosters cell survival by means of its physical interaction with and inhibition of the MAP JNK kinase, mitogen-activated protein kinase kinase 7 (MKK7, Uniprot AC: O14733; 419 amino acids; Molecular mass 47,485 Da), an essential activator of the pro-apoptotic JNK signalling pathway [[2], [3], [4], [5]]. Accordingly, the GADD45β/MKK7 complex is an essential, cancer cell-restricted survival module downstream of the NF-κB pathway. Moreover, MM patients exhibiting high levels of GADD45β expression had reduced progression-free survival (PFS) and overall survival (OS) rates compared to patients expressing lower GADD45β levels, demonstrating that elevated GADD45β expression denotes more aggressive disease and a has less favourable clinical outcome. On the basis of these findings, we reasoned that therapeutically targeting the GADD45β/MKK7 complex would selectively block the NF-κB survival function in MM cells, without affecting the pleiotropic and ubiquitous physiological functions of NF-κB.

By screening about 20,000 different tetrapeptides, we identified some lead compounds that blocked the interaction between GADD45β and MKK7 in a highly selective manner, without compromising the catalytic activity of MKK7. We next developed the D-tripeptide, DTP3 (Ac-D-Tyr-D-Arg-D-Phe-NH_2_), which bound to MKK7 with high affinity and disrupting the GADD45β/MKK7 interaction, thereby restoring the MKK7 kinases activity and, ultimately, the JNK-mediated cell death pathway. As a result of this therapeutic mode of action, DTP3 was highly effective in killing MM cell lines and primary cancer cells from MM patients *in vitro*, with far greater cancer cell specificity than IκBα kinase (IKK)β/NF-κB inhibitors. Similarly, upon intravenous infusion, DTP3 caused a marked regression of MM in mouse xenograft models *in vivo*, with good tolerability and no apparent adverse effects. However, DTP3 is not suitable for oral administration, which could be highly advantageous for its broad clinical use. Therefore, its further development into a second-generation non-peptidic compound would require a detailed understanding of the molecular interaction between GADD45β and MKK7, as well that between DTP3 and MKK7, its molecular target.

Previous studies have shown that GADD45β effectively binds to and inhibits MKK7 [2,4,6], preventing the prolonged activation of JNK signalling and ultimately cell death. Although molecular models of the GADD45β/MKK7 complex have previously been reported [2,4,6], the exact molecular mechanism by which GADD45β interacts with and blocks MKK7 remains elusive, due to the lack of experimental structural data on this complex. The GADD45β/MKK7 interaction has been largely investigated by the use of mutagenesis experiments [4,5] in parallel with molecular modelling studies. These studies suggested that the complex between the two proteins is mediated by a network of H-bonds between residues located at the complex interface and by further electrostatic interactions between the so-called acidic loop 1 (residues 61–68) of GADD45β and basic residues in the MKK7 catalytic pocket, including the functionally critical catalytic residue, Lys149. According to this model, GADD45β physically hindered the access of ATP to the catalytic pocket of MKK7, thereby blocking its enzymatic activity.

On the basis of the crystallography and NMR data available for the kinase domain of MKK7 [[7], [8], [9]] and for the GADD45β homologues, GADD45α and GADD45γ [10,11], the structure of the GADD45β/MKK7 complex has been re-examined in a recent molecular modelling study [2], which while confirming that the interaction with GADD45β prevents the access of ATP to the MKK7 catalytic pocket, has suggested that this interaction is mediated by the GADD45β so-called loop 2 (residues 103–117), which, like loop 1, is rich in acidic residues, but is longer and more flexible. This new study also predicts that the structure and stoichiometry of the complex might be complicated by the occurrence of homodimers of both GADD45β and MKK7 [2]. Our previous study demonstrated that DTP3 works by binding to MKK7 and inducing a conformational change of the kinase that is likely responsible for the dissociation of GADD45β. Although there is information on the pharmacophore that binds to MKK7, the precise molecular details and mechanism underlying the DTP3/MKK7 interaction and the kinase conformational rearrangement remain poorly understood. Therefore, in order to support the clinical development of DTP3, it is important to progress the understanding of the structural basis for the GADD45β/MKK7 interaction and the mode of action of DTP3.

Using a MS-based approach, which is widely recognized as a powerful tool for resolving the structural features of protein complexes, we investigated the protein surfaces involved in the GADD45β/MKK7 interaction in order to elucidate amino acidic regions involved in protein-protein recognition and identify the residues of MKK7 making contacts with DTP3. To this end, we used enzymatic MS-based foot-printing and chemical cross-linking MS (CX-MS) analyses. The results provide important new structural information on the GADD45β/MKK7 protein complex and provide fundamental new insights into the interaction of DTP3 with MKK7, with profound implications for the design of second-in-class, small-molecule GADD45β/MKK7 inhibitors.

## 2 Materials and methods

### 2.1 Preparation and characterization of recombinant proteins

Recombinant His6-GADD45β (hereafter named GADD45β) and His6-MKK7 Kinase Domain (MKK7_KD, residues 101–405, hereafter named MKK7_KD) were prepared as previously reported [2,6]. Briefly, recombinant proteins were expressed in *E. coli*, BL21(DE3) strain, using the pETDuet-1 and the pET28a vectors (Novagen) for MKK7_KD and GADD45β, respectively. After lysis, proteins were purified by affinity chromatography using a His-Trap column (GE Healthcare), followed by a gel filtration step on a Superdex 75 10/30 Gel Filtration column. Purified proteins were characterized by 12% SDS-PAGE, analytical Size Exclusion Chromatography (SEC), Light Scattering and MALDI-TOF mass spectrometry to assess purity and identity and by Circular Dichroism (CD) to assess proper folding. Details of these characterizations are reported in [2,6,12].

Binding of DTP3 (Ac-D-Tyr-D-Arg-D-Phe-NH_2_) to MKK7_KD was assessed by tryptophan fluorescence quenching experiments for the interaction of full-length GST-fused MKK7 with the same tripeptide [2]. Spectra were acquired with a Cary Eclipse Spectrofluorimeter (Varian Inc.), equipped with a Peltier thermostating system, and a 1-cm quartz cuvette. The analyses were carried out using solutions at 1.25 μM of MKK7_KD dissolved in 10 mM phosphate buffer pH 7.5, in the presence of increasing concentrations of DTP3 or control scrambled D-peptide (Ac-D-Arg-D-Phe-D-Tyr-NH_2_, SCRB) dissolved in the same buffer. 1 μL aliquots of peptide solutions at increasing concentrations (between 0.6 μM and 296 μM) were added to the cuvette (400 μL). Data were acquired at 25.0 °C, using an excitation wavelength of 295.0 nm, in order to avoid interference with tyrosine residues, within the fluorescence emission wavelength range 300–450 nm. The excitation and emission slits were set at 5 nm, the scan rate was 120 nm/min, the data interval was 1.00 nm, the averaging time was set at 0.500 s, the PMT voltage was set at “high.” All the spectra were smoothed using the same algorithm. The fluorescence emission values recorded at 333 nm were extracted, transformed to -ΔFluorescence and, then, plotted against the peptide concentration to estimate the K_D_ (Dissociation constant) related to the peptide/kinase interaction.

To further assess the function of the recombinant protein, the binding of MKK7_KD to GADD45β was verified by ELISA and by Surface Plasmon Resonance (SPR) using a Biacore3000 instrument. Experimental conditions have been reported in Tornatore et al. [2]. GADD45β was covalently immobilized on the surface of a CM5 Biacore sensorchip at pH 3.5 following the canonical EDC/NHS methodology [12]. ELISA tests were performed coating MKK7_KD on the surface of polystyrene plate wells at 100 nM and using biotinylated GADD45β at concentrations between 10 nM and 160 nM. Detection was achieved by using Streptavidin conjugated with HRP, under the conditions reported in Tornatore et al. [2].

A shorter variant of the MKK7_KD corresponding to the protein fragment 137–405 was also prepared to investigate the role of tryptophan 135 on the quenching effects produced by the interaction of DTP3 with the kinase. This variant was designed because of the presence of a starting methionine on position 137. The cDNA corresponding to this fragment was amplified by PCR (Tm = 70.8 °C) from the MKK7_KD cDNA using the following primers: MKK7_KD 137–405 Forward T1 (GCGCGCGGATCCAATGCGCTTCCGGAAGACC) and MKK7_KD 137-405 Reverse T1 (CGCGCGGAATTCTTATCACCGCGGTGACTCAGTCTTCG). The resulting DNA fragment amplified by PCR was cloned into the BamHI and EcoRI sites of the pETDuet plasmid and the corresponding protein, bearing a His6 tag by way of the plasmid construct, was expressed in *E. coli* under the same conditions reported for the preparation of the 101–405 domain. The protein was recovered from the inclusion bodies, dissolved in 6 M Urea and refolded by gradual dilution of the denaturing agent up to 0.5 M. The protein in 50 mM phosphate buffer pH 7.3, 0.5 M Urea was purified by affinity chromatography on a HisTrap column and by gel filtration chromatography and was subsequently characterized by 12% SDS-PAGE and CD at a concentration of 4 μM. Binding of DTP3 to this domain was monitored by tryptophan fluorescence quenching experiments, performed as described for the MKK7_KD 101–405 domain, except for the buffer which was 10 mM phosphate buffer pH 7.0 containing 5 mM DTT and 0.5 M Urea. Fluorescence quenching experiments were repeated in this buffer also with MKK7_KD 101–405. Protein concentration was 1.25 μM. Since the protein was somehow less soluble than the longer variant, CD experiments were performed in a different buffer: 20 mM Tris pH 7.5, 100 mM NaCl, containing 5 mM DTT and 0.5 M Urea.

### 2.2 Enzymatic MS-based foot-printing

For MS-based foot-printing analyses, tryptic hydrolyses were performed by adding bovine TPCK-treated trypsin (1 μg/μL, Sigma) to aliquots (1 nmol in 20 mM Tris-HCl pH 7.5, 100 mM NaCl, 1 mM DTT) of GADD45β and MKK7_KD alone or in combination to obtain the complex GADD45β/MKK7_KD at an enzyme/substrate ratio of 1:100 (w/w) in a final volume of 100 μL and by incubating the mixtures overnight at 37 °C. Similar experiments were carried out in the presence of a 5-fold mol/mol excess of DTP3. Protein digestions were blocked by 1:5 dilution in 0.1% formic acid in water.

Peptide mixtures were analyzed by using an Acquity H-Class UPLC coupled with a Xevo TQD triple quadrupole mass spectrometer equipped with an ESI source (Waters). Aliquots of the tryptic digest (20 pmoles in 10 μL) were loaded on an Acquity HSS T3 column packed with a trifunctional C_18_ alkyl phase (100 mm length × 2.1 mm ID, 1.8 μm particle size), maintained at 40 °C (Waters). The optimized mobile phase consisted of 98% water/2% CH_3_CN/0.1% formic acid (solvent A) and 98% CH_3_CN/2% water/0.1% formic acid (solvent B). Chromatographic separations were performed by using a gradient from 5% to 55% of buffer B over 60 min at a flow rate of 0.3 mL/min. The triple quadrupole operated in the positive mode between *m/z* 300 and 2000. The ESI source operating parameters were set as follows: positive ionization mode; capillary voltage 3.9 kV; source temperature 150 °C; desolvation temperature 500 °C; desolvation nitrogen gas flow rate 1000 L/h; sampling cone voltage 39 V.

LC-MS data were analyzed by integrating peaks of the extracted ion chromatograms of peptide precursor ions. The areas of each chromatographic peak were then normalized on the total areas of their respective mass chromatograms. Subsequently, the ratio of peak areas in the analysis of GADD45β/MKK7 complex *versus* the areas of the same peaks obtained by digesting the isolated proteins was calculated.

### 2.3 Chemical cross-linking reactions

#### 2.3.1 Cross-linking of the GADD45β/MKK7_KD complex

Samples (100 μg) of GADD45β and MKK7_KD were dissolved in 10 mM Sodium Phosphate buffer pH 8.0 to a final concentration of 10 μM. The amine-reactive cross-linking reagent NHS-Diazirine (SDA) was used as photoactivable reagent (Pierce Biotechnology, ThermoFisher). Preliminary experiments were carried out to determine the optimal protein concentrations and cross-linking reagent-to-protein molar ratios. Following the optimization of the reactions, SDA was dissolved in 10 mM DMSO pH 8.0 and added in a first step to the MKK7_KD protein solution (270 μL) with a 20-fold excess (mol/mol) of the cross-linker. Following incubation for 1 h at room temperature, reactions were quenched by adding 1 M Tris-HCl buffer pH 8.0 at a final concentration of 50 mM. Then, the unreacted cross-linker was removed by microfiltration by using Corning Spin-X UF Concentrators MWCO 5000 (Millipore). Protein quantification was performed by Bradford assay. For the second step, equal moles (700 pmol) of GADD45β were added to the SDA-labeled MKK7_KD to perform the UV-cross-linking reaction (40 μL final volume). Protein mixtures in clear, V-bottom 96-well plates were irradiated at 365 nm for 15 min on ice by using a Black-Ray UVP B-100 A, 100 W lamp (UVP).

#### 2.3.2 Cross-linking of the DTP3/MKK7_KD complex

A non-acetylated variant of DTP3 (H-D-Tyr-D-Arg-D-Phe-NH_2_) was dissolved in dry DMSO pH 8.0 at a final concentration of 8.25 mM. A freshly prepared solution of SDA cross-linker in DMSO pH 8.0 (15 mg/mL) was then incubated with the compound solution in 1:1 M ratio. Reaction of NHS ester was monitored by analytical HPLC on a ONYX Monolithic C18 column (50 × 2 mm, Phenomenex) at a flow rate of 0.6 mL/min by using a gradient from 2 to 60% in 6 min (solvent A 0.05% TFA in water, solvent B 0.05% TFA in acetonitrile). Following 90 min incubation at room temperature, the resulting product was purified by preparative RP-HPLC on a C18 column (100 × 10 mm, ONYX Monolithic, Phenomenex) at a flow rate of 10 mL/min by using a gradient from 2 to 60% in 10 min (buffer A 0.08% TFA in water, buffer B 0.08% TFA in acetonitrile). SDA-labeled DTP3 was analyzed by ESI-MS on a Ion Trap (Bruker) by direct infusion. Upon lyophilisation, the product was dissolved in Milli-Q water at a final concentration of 2 mM. For the photo-labeling step, MKK7_KD (700 pmol, 5.5 μM in sodium phosphate buffer pH 8.0) was incubated for 15 min at room temperature in the dark with the SDA-labeled DTP3 with a 10-fold excess (mol/mol) of the peptide. Equal amounts of MKK7_KD without the labeled peptide were in parallel analyzed to evaluate the specificity of the reaction. Then, for the UV-cross-linking reaction (50 μL final volume) the diazirine group was photo-activated by UV irradiation at 365 nm for 15 min on ice as described above. Removal of unreacted SDA-labeled DTP3 and buffer exchange to 50 mM ammonium bicarbonate pH 7.8 were performed using Corning Spin-X UF Concentrators MWCO 5000 (Millipore).

#### 2.3.3 In solution tryptic digestion

Cross-linked samples in 10 mM sodium phosphate buffer pH 8.0 for the GADD45β/MKK7_KD complex or in 50 mM ammonium bicarbonate buffer pH 8.0 for the DTP3/MKK7_KD complex were reduced at 55 °C for 1 h by adding dithiothreitol (DTT) up to 10 mM final concentration. Following carbamidomethylation with Iodoacetamide (IAM, 7.5 mM final concentration) at room temperature in the dark for 15 min, enzymatic hydrolyses were performed with TPCK-treated bovine trypsin (Sigma Aldrich) at 37 °C for 24 h with an enzyme-to-substrate ratio of 1:50 (w/w). After digestions, samples were centrifuged at 10.000 ×*g* for 15 min and supernatants were diluted in H_2_O/Formic acid 0.1% at a final concentration of 500 fmol/μL for MS analysis.

#### 2.3.4 *In situ* tryptic digestion

In addition to the tryptic digestion in solution, an *in situ* tryptic digestion was performed on the cross-linked DTP3/MKK7_KD complex. To this aim, following photo-activation, the reaction products were resolved by 12% SDS-PAGE. Protein bands were excised from gels and de-stained by performing two washes with 200 μL of water and then with 50% acetonitrile. Gel pieces were dried in a SpeedVac Vacuum (Savant Instruments) and rehydrated with 50 μL of 50 mM ammonium bicarbonate (NH_4_HCO_3_) pH 8.0. Samples were reduced at 55 °C for 15 min by adding DTT up to 10 mM final concentration and alkylated for 15 min in the dark at room temperature with IAM (40 mM final concentration). Following two washes with 200 μL of water and then with 50% acetonitrile, gel pieces were dried and rehydrated with 50 μL of 50 mM NH_4_HCO_3_ pH 8.2. Enzymatic digestions were performed by incubation at 37 °C for 3 h following the addition of 1 μL of a 140 ng/μL TPCK-treated bovine trypsin solution (Sigma Aldrich). Peptides were extracted in two steps by sequential addition of 50 μL of 1% TFA and then 50 μL of 2% TFA/50% acetonitrile for 10 min in a sonication bath. The combined supernatants were dried in the SpeedVac Vacuum and resuspended in 5 μL of 0.1% TFA/50% acetonitrile. Samples were diluted 1:20 in 0.1% TFA/H_2_O and centrifuged at 10.000 ×*g* for 15 min. Aliquots of the supernatant (5 μL) were analyzed by nanoLC−Tandem Mass Spectrometry as described in the Section 2.3.5.

#### 2.3.5 Identification of cross-linked products by high-resolution nanoLC−Tandem Mass Spectrometry

Cross-linked samples prepared as described above, were analyzed by high-resolution nanoLC−Tandem Mass Spectrometry using a Q Exactive Orbitrap mass spectrometer equipped with an EASY-Spray nano-electrospray ion source (Thermo Fisher Scientific) and coupled to a Thermo Scientific Dionex UltiMate 3000RSLC nano system (Thermo Fisher Scientific). Solvent composition was 0.1% formic acid in water (solvent A) and 0.1% formic acid in acetonitrile (solvent B). Peptides were loaded on a trapping PepMap™100 μ Cartridge Column C18 (300 μm × 0.5 cm, 5 μm, 100 Å) and desalted with solvent A for 3 min at a flow rate of 10 μL/min. After trapping, eluted peptides were separated on an EASY-Spray analytical column (15 cm × 75 μm ID PepMap RSLC C18, 3 μm 100 Angstrom and 50 cm × 75 μm ID PepMap RSLC C18, 3 μm, 100 Angstrom for samples from in-solution and *in situ* digestions, respectively), heated to 35 °C, at a flow rate of 300 nL/min by using the following gradient: 4% B for 3 min, from 4% to 22% B in 50 min, from 22% to 35% B in 10 min, from 35% to 90% B in 5 min. A washing (90% B for 5 min) and a re-equilibration (4% B for 15 min) step was always included at the end of the gradient.

Eluting peptides were analyzed on the Q-Exactive mass spectrometer operating in positive polarity mode with capillary temperature of 280 °C and a potential of 1.9 kV applied to the capillary probe. Full MS survey scan resolution was set to 70,000 with an automatic gain control (AGC) target value of 3 × 10^6^ for a scan range of 375–1500 *m/z* and maximum ion injection time (IT) of 100 ms. The mass at *m/z* 445.12003 was used as lock mass. A data-dependent top 5 method was operated during which higher-energy collisional dissociation (HCD) spectra were obtained at 17500 MS2 resolution with AGC target of 1 × 10^5^ for a scan range of 200–2000 *m/z*, maximum IT of 55 ms, 2 *m/z* isolation width and normalized collisional energy (NCE) of 27. Precursor ions targeted for HCD were dynamically excluded for 15 s. Full scans and Orbitrap MS/MS scans were acquired in profile mode, whereas ion trap mass spectra were acquired in centroid mode. Charge state recognition was enabled by excluding unassigned and singly charge states.

Acquired raw files were analyzed by the Thermo Scientific Proteome Discoverer 2.1 and the Thermo Xcalibur 3.1 (Thermo Fisher Scientific) software. Cross-linked peptides were identified by manual searches and with the support of the StavroX software (v. 3.6.0.1) [13] with the following parameter settings: protease sites: K, R; missed cleavages sites: K = 3, *R* = 2; fixed modification: carbamidomethylation (B); variable modifications: max 1 methionine oxidation (m); cross-linker: SDA (C_8_O_1_H_6_); cross-linked site 1: K, S, T, Y, N-term; site 2: A, I, L, M, S, T, W, H, D, E, N, K, P, G, V, Q, m, C, B, Y, R, F. Precursor mass deviation: 10 ppm; Fragment mass deviation: 0.02 Da; lower mass limit: 200 Da; upper mass limit: 6000 Da; S/N ratio: 2.0; ion types: b-and y-ions; no neutral loss. Extracted ion chromatograms of signal ions corresponding to the cross-linked peptides were manually generated by the Thermo Xcalibur 3.1 software.

### 2.4 Model structure of GADD45β/MKK7_KD complex

The model of the GADD45β/MKK7_KD complex is the one reported in Tornatore et al. [2]. Details for its construction and validation are reported in the same paper [2]. Briefly, we applied the protein-protein docking procedure implemented in the ClusPro 2.0 web server in order to obtain a GADD45β/MKK7 complex model with default parameters. The model was built from the model of GADD45β and MKK7, taking into account data from site-directed mutagenesis and *in vitro* binding studies [4]; the following amino acid residues were used as restraints during the docking procedure: MKK7, residues Val134, Trp135, Lys136, Arg138, Lys149, and Arg162; GADD45β, residues Glu63, Glu64, Asp67, Asp68, His74, Gln109, Thr111, Trp112, Glu113, Arg115, and Leu117. The centroid of the largest cluster of solutions was selected as the best model of the complex and was refined in explicit solvent using the HADDOCK web server as described in [2]. To identify regions involved in protein-protein and peptide-protein interactions, the structure was visualized by Chimera software (www.cgl.ucsf.edu/chimera [14]).

## 3 Results

### 3.1 Expression and purification of recombinant proteins

Recombinant GADD45β was prepared as previously described [6]. Interaction studies of DTP3 with MKK7 were performed using the MKK7_KD protein kinase domain [2], since previous studies indicated that both GADD45β and the tripeptide recognize this enzyme region [2,4,6]. We chose the region encompassing residues 101–405 with the Ser271Asp and Thr275Asp mutations, on the basis of previous structural studies showing that it was folded and stable enough to be crystallized and studied by X-Ray diffraction (PDB ID: 2DYL). The indicated serine and threonine residues, which are phosphorylated in the activated protein, were replaced with aspartic acid to obtain a constitutively active enzyme.

After purification by affinity and gel filtration chromatography, proteins were obtained with a purity level >95%, as estimated by SDS-PAGE (Supplementary material Fig. S1A) and mass spectrometry (Supplementary material Fig. S1B–C). In detail, the presence of single bands at about 37 kDa and 20 kDa was consistent with the expected molecular weight for MKK7_KD and GADD45β, respectively. The experimental molecular masses determined by MALDI-TOF mass spectrometry were also consistent with those calculated on the basis of the primary structure for both GADD45β (Theoretical mass: 19196.6 Da/Experimental mass: 19204.1 Da, Supplementary material Fig. S1B) and MKK7_KD (Theoretical mass: 36194.8 Da/Experimental mass: 36251.2 Da, Supplementary material Fig. S1C). SEC characterization of MKK7_KD gave one single peak at an elution volume of 10.6 mL and light scattering analysis showed a molecular mass of about 37 kDa consistent with the expected molecular mass and demonstrated that the protein was monomeric (data not shown).

### 3.2 Characterization of MKK7_KD

MKK7_KD was characterized by CD and for its ability to bind to GADD45β. The CD spectrum of the protein (Supplementary material Fig. S2A) was consistent with the protein secondary structure content and with that previously reported [2]. Experiments of tryptophan fluorescence quenching were performed to estimate the affinity of the peptide with the protein. Fluorescence emission spectra of the protein upon excitation at 295 nm, in absence and in the presence of increasing amounts of both DTP3 and the scrambled peptide are shown in Supplementary material Fig. S2B–C. By plotting the maximum values at 333 nm *versus* peptide concentration, the saturation curve reported in Supplementary material Fig. S2D was obtained, from which a dissociation constant (K_D_) of about 100 nM was derived.

Binding of MKK7_KD to GADD45β was next assessed through Biacore and ELISA experiments. In Supplementary material Fig. S3A an overlay of 6 sensorgrams obtained by injection of the soluble MKK7_KD onto a sensor chip with immobilized GADD45β is reported. An estimation of the K_D_, obtained by averaging the different values determined at the various concentrations, gave a value of about 6 nM similar to that reported for the full-length protein [2,6].

Binding of MKK7_KD to GADD45β was also performed using a biotinylated variant of GADD45β. The dose-dependent values obtained at MKK7_KD concentrations between 0 and 150 nM are plotted in Supplementary material Fig. S3B. As shown, binding was dose-dependent and saturated at biotin-GADD45β concentrations higher than about 150 nM. The K_D_ was estimated by a curve fitting mathematical 1:1 model which provided a value of about 30 nM.

### 3.3 Enzymatic MS-based foot-printing for the identification of the GADD45β/MKK7 interaction surface

Although molecular models of the GADD45β/MKK7 interaction have been predicted [4,5,15], the interaction surfaces of the complex have not been yet fully elucidated and no experimental structural studies are available to date on the protein complex. To investigate the interaction surfaces of the GADD45β/MKK7 complex, we probed the accessible surfaces of single proteins alone and in complex in the presence and absence of DTP3 by MS. By this approach, protection against proteolysis is used to map interaction sites in protein complexes. In a typical protein foot-printing experiment, the complex is incubated under native conditions with the protease. MS analysis of the resulting peptide mixtures allows the identification of regions protected from protease activity in comparison with peptide mixtures from enzymatic hydrolysis of single proteins [16,17]. These experiments can be also conducted in the presence and absence of a specific ligand to map ligand-induced changes on proteolytic fragments.

At first, isolated proteins were digested with trypsin under native conditions and the resulting peptides were analyzed by LC-MS as described in the Methods section. LC-MS chromatograms of the tryptic peptides obtained from GADD45β and MKK7_KD digestions are shown in Supplementary material Fig. S4 and Fig. S5, respectively. These elution profiles are also reported in comparison with the LC-MS chromatogram of peptides derived from the tryptic digestion of the GADD45β/MKK7_KD complex in Supplementary material Fig. S6. Peptides mapped on GADD45β and MKK7_KD are reported in Supplementary material Table S1A–B. Extracted ion chromatograms of peptide precursor ions were integrated and normalized as described in the Methods section in order to compare peak areas in GADD45β/MKK7 complex with respect to isolated proteins (Supplementary material Table S2A–B). By this approach, we found that most tryptic peptides obtained under native conditions were not affected by complex formation. Nevertheless, the G_T8 and M_T8 peptides were protected from proteolysis in the protein complex. In particular, a ≈3-fold decrease of intensity was found for the triply charged ion at *m/z* 622.3, eluting at retention time 27.2 min for GADD45β and that of the doubly charged ion at *m/z* 484.3, eluting at retention time 23.8 min for MKK7_KD. These peptides correspond to the G_T8 peptide of GADD45β (116-DLHCLLVTNPHTDAWK-131, precursor ion mass 1861.9 Da) and to the M_T8 peptide of MKK7_KD (259-LCDFGISGR-267, precursor ion mass 966.5 Da). Interestingly, both peptides are localized on the outer surface of the respective proteins (Supplementary material Fig. S7) and fall within regions identified as the interaction surface by previous studies based on structural predictions [2].

MS-based foot-printing experiments performed in the presence of the inhibitor DTP3 showed that the N-terminal region 102–112 of the kinase domain was strongly protected from trypsin cleavage when the protein is in complex with GADD45β or interacts with the tripeptide (Supplementary material Table S3; see peptide M_T4), suggesting that it might be the one undergoing the conformational rearrangement observed by CD analysis [2]. Indeed, M_T4 intensity is substantially unaffected in the presence and in the absence of GADD45β alone (Supplementary material Table S2B; see peptide M_T4), while it largely decreases in the presence of DTP3 alone and in the presence of both GADD45β and DTP3.

### 3.4 Chemical cross-linking of the GADD45β/MKK7_KD complex

**Fig. 1:**
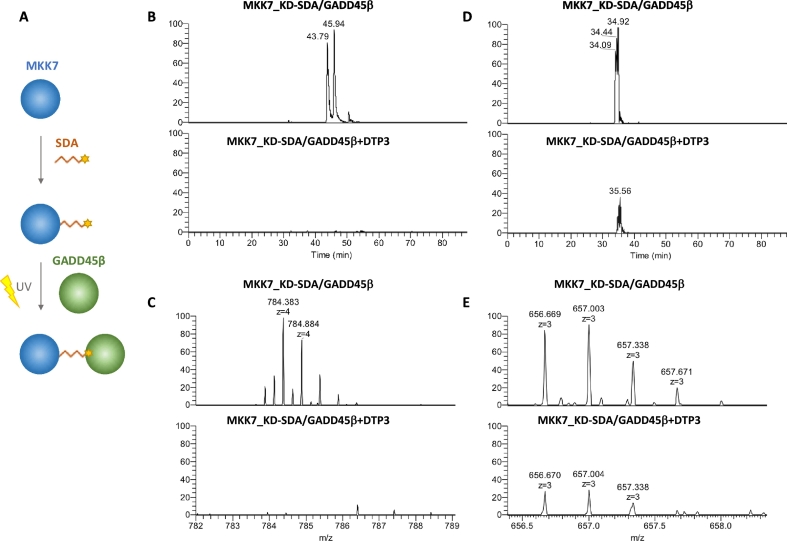
(A) Schematic workflow showing the key steps of the photo-chemical cross-linking reactions used for mapping the interaction surfaces of the GADD45β/MKK7_KD complex. (B–D) Extracted ion chromatograms of the quadruply charged ion at *m/z* 784.384 (C) and of the triply charged ion at *m/z* 656.699 (E) of the cross-linking reactions performed in the absence and in the presence of DTP3.

To validate the results from MS-based foot-printing, we investigated the protein regions involved in the GADD45β/MKK7_KD complex interaction by applying a chemical photo-cross-linking approach. We used the hetero-bifunctional reagent, succinimidyl 4,4′-azipentanoate (SDA), containing an amine-reactive N-hydroxysuccinimide (NHS) ester and a photoactivatable diazirine group at the end of a short spacer arm (3.9 Å). We found that when SDA was first reacted with GADD45β, only its homo-dimers were detected likely due to an increased propensity of GADD45β for oligomerization compared to binding with MKK7 [6]. Therefore, following an optimization of the reaction conditions (see Section 2.3.1), the hetero-dimer GADD45β/MKK7_KD was obtained by performing first the NHS-reaction on MKK7_KD followed by addition of GADD45β and UV cross-linking (Fig. 1A). Following photo-activation, samples were digested with trypsin and the resulting peptide mixtures were analyzed by high-resolution nanoLC-MS/MS.

Using this approach, we identified two pairs of intermolecular cross-linked peptides, which were representative of the GADD45β/MKK7_KD interaction sites. The first cross-linked peptide pair, identified by MS/MS fragmentation of the quadruply charged ion at *m/z* 784.384 (theoretical molecular mass of the precursor ion 3133.540 Da), eluted at 43 min and involved a fragment adjacent to the acidic loop 2 of GADD45β and the C-terminus of MKK7_KD. This intermolecular cross-link occurred between peptide 396–405 of MKK7_KD and peptide 116–131 of GADD45β (Fig. 1B–C), consistent with the results from previous MS-based foot-printing experiments. The second interaction site, which was identified by the MS/MS spectra of the triply charged ion at *m/z* 656.669 (theoretical molecular mass of the precursor ion 1966.997 Da), eluted at 35 min and corresponded to the cross-link between peptide 92–97 of GADD45β and peptide 102–112 of MKK7_KD (Fig. 1D–E). The MS/MS spectra of the quadruply charged ion at *m/z* 784.384 and the triply charged ion at *m/z* 656.699 corresponding to the detected intermolecular cross-links are reported in Supplementary material Fig. S8 and Fig. S9, respectively.

Both the identified cross-linked peptide pairs are localized at the GADD45β/MKK7_KD complex interface, in agreement with the molecular 3D model *in silico* predicted for the complex (see Discussion). Significantly, these were the only cross-linked peptides observed in this experiment.

Interestingly, when the same cross-linking reactions were performed in the presence of DTP3, we did not observe the cross-linked peptide pair MKK7_KD 396-405/GADD45β 116-131 (Fig. 1B–C), while the peptide pair MKK7_KD 102-112/GADD45β 92-97 was detected with a markedly decreased intensity (Fig. 1D–E). These results are in keeping with the ability of DTP3 to cause the dissociation of the GADD45β/MKK7 protein complex.

### 3.5 Identification of the DTP3 interaction site on MKK7_KD

**Fig. 2:**
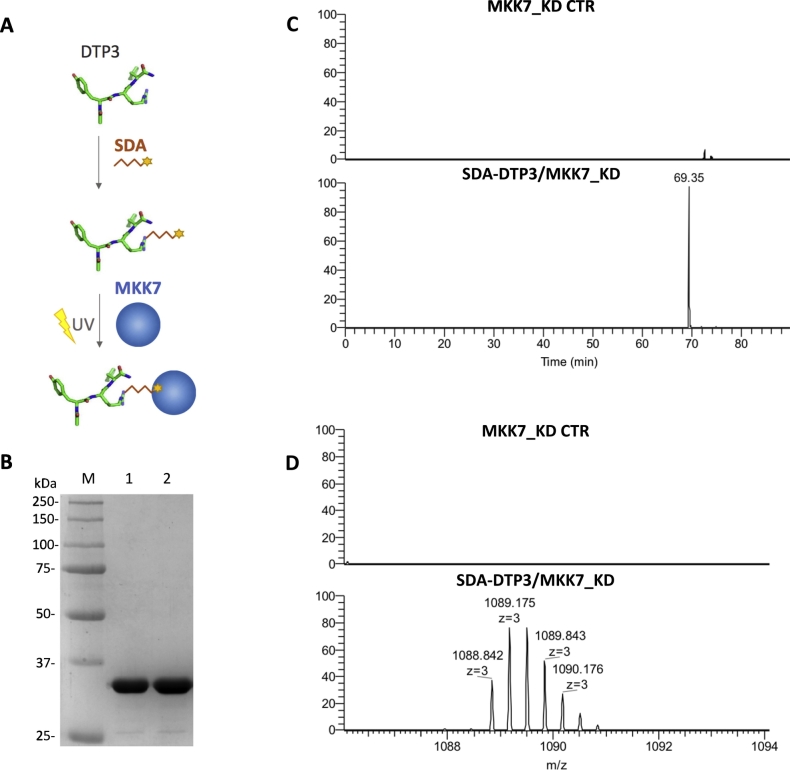
(A) Schematic workflow showing the key steps of the photo-chemical cross-linking reactions used for mapping the DTP3 interaction site on MKK7_KD. (B) SDS–PAGE analysis (12% resolving gel) of the SDA-DTP3/MKK7_KD cross-linked complex (Lane 1 MKK7_KD negative control; Lane 2 MKK7_KD incubated with SDA-DTP3; M, protein markers). (C) Extracted ion chromatograms of the triply charged ion at *m/z* 1088.842 (D) of the cross-linking reactions performed in the absence and in the presence of SDA-DTP3 followed by *in situ* tryptic digestion and high-resolution nanoLC-MS analysis.

Although previous studies have reported that DTP3 interacts with the kinase domain of MKK7, the amino acidic region mediating this interaction remains to be mapped. Therefore, a similar chemical cross-linking mass spectrometry strategy was also used to investigate the site(s) of MKK7_KD that interact with DTP3. We therefore used DTP3 in the first step of the NHS-reaction, followed by the addition of MKK7_KD to enable the UV cross-linking reaction (Fig. 2A). The DTP3 labeling reaction with SDA was monitored by analytical HPLC (Supplementary material Fig. S10A). The final product of the reaction, that is SDA-DTP3, was purified by preparative RP-HPLC, resulting in a yield of about 63%, as determined by mass spectrometry analysis (Supplementary material Fig. S10B–C). In the second step of the reaction, SDA-DTP3 was incubated with MKK7_KD to enable the UV cross-linking (Fig. 2A). A MKK7_KD protein incubated in the absence of SDA-DTP3 was used in a parallel photo-activation experiment as negative control. Following photo-activation, samples were separated by SDS-PAGE (Fig. 2B), and the resulting gel bands were excised, subjected to enzymatic *in situ* tryptic digestion, and analyzed by high-resolution nanoLC-MS/MS. Using this experimental approach, we identified a triply charged ion at *m/z* 1088.842, corresponding to a cross-linked chemical product consisting of peptide 113–136 of MKK7_KD and DTP3, connected by the short linker originating from SDA (Fig. 2C–D).

**Fig. 3:**
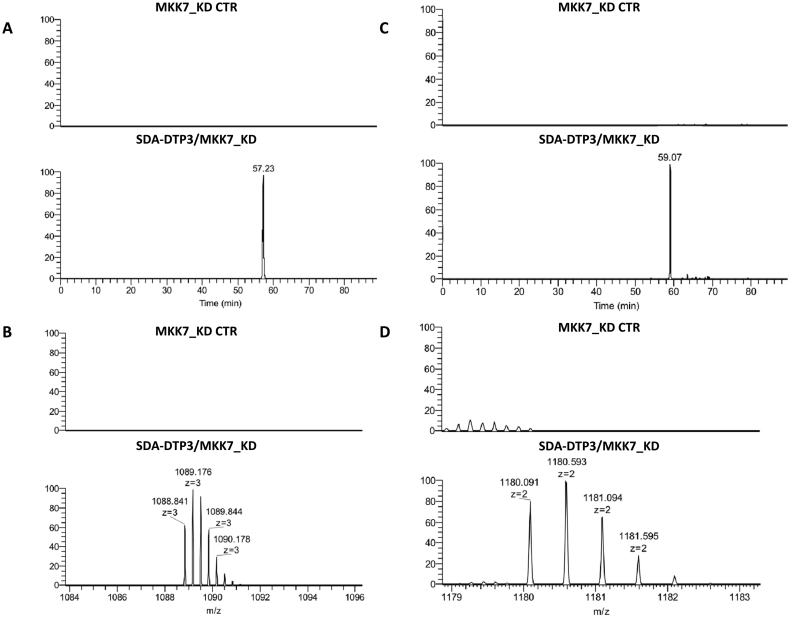
(A–C) Extracted ion chromatogram of the triply charged ion at *m/z* 1088.841 (B) and of the doubly charged ion at 1180.091 (D), respectively, of the cross-linking reactions performed in the absence and in the presence of SDA-DTP3 followed by in solution tryptic digestion and high resolution nanoLC-MS analysis.

To increase the confidence level in the cross-linked product identification, we performed a shot-gun LC-MS analysis of tryptic peptides obtained by in-solution digestion on the photo-activated SDA-DTP3/MKK7_KD sample. Using this approach, we confirmed the presence of the cross-linked product comprising DTP3 and fragment 113–136 of MKK7_KD, previously identified by the *in situ* tryptic digestion. The quadruply charged ion of the cross-linked peptide pair is shown in Fig. 3A–B (*m/z* 1088.841). In this experiment, we also identified an additional cross-linked product consisting of DTP3 and peptide 259–274 of MKK7_KD, which was absent in the negative control experiment and detected as a doubly charged ion at *m/z* 1180.091 (Fig. 3C–D). It is worth noting that the N-terminal peptide of MKK7 which was cross-linked with DTP3 contained the tryptophan residue located at position 135. Consistent with this finding and the contact of DTP3 with a protein region responsible for tryptophan fluorescence emission, an effect of intrinsic fluorescence quenching was consistently observed upon the interaction of DTP3 with MKK7 [2] or with its isolated kinase domain (see also Supplementary material Fig. S2B–D).

We thus sought to further validate the DTP3/MKK7_KD interaction by performing a fluorescence quenching study on an N-terminally truncated MKK7 kinase domain protein lacking the amino acidic region 101–136.

**Fig. 4:**
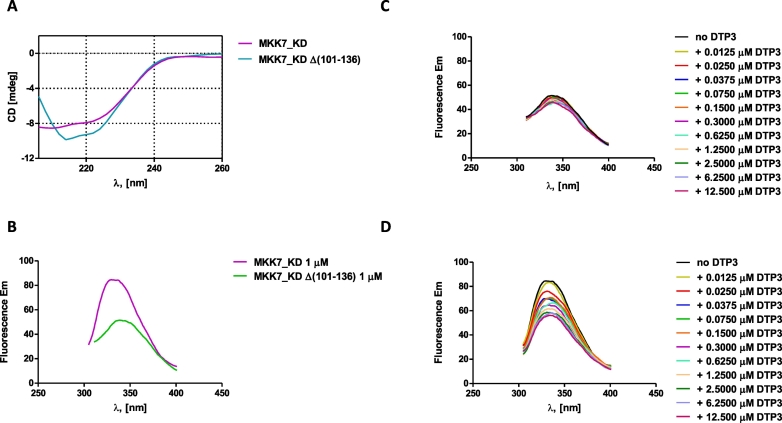
(A) CD spectra of MKK7_KD and of MKK7_KD Δ(101–136). The spectrum of the shorter protein could be only collected up to about 200 nm due to the presence of NaCl, DTT and urea. (B) Fluorescence spectra of MKK7_KD (magenta) and of the shorter protein (green). Fluorescence emission spectra of recombinant MKK7_KD (101–405) (C) and MKK7_KD (137–405) (D) in the presence of increasing concentrations of DTP3.

The MKK7_KD Δ(101–136) polypeptide was expressed in *E. coli*, purified to homogeneity (data not shown), and characterized by CD to verify that it was properly folded. The CD spectrum of the MKK7_KD Δ(101–136) protein is shown in Fig. 4A, where it is compared to the spectrum of MKK7_KD. These data demonstrate that the MKK7_KD Δ(101–136) protein is folded and exhibits a prevailingly alpha helical content, as expected on the basis of its protein structure. However, its spectrum was not fully comparable to that of the MKK7_KD 101–405 variant due to the presence of DTT and urea used to improve its solubility. Next, we verified the binding of DTP3 to both MKK7_KD and MKK7_KD Δ(101–136) by means of tryptophan fluorescence quenching analysis, using the same buffer as for the MKK7_KD shorter domain in order to directly compare the results. The fluorescence emission spectra of both proteins, upon excitation at 295 nm, are reported in Fig. 4B, while the overlaid emission spectra obtained upon DTP3 addition at the indicated concentrations are shown in Fig. 4C and Fig. 4D. Data demonstrate that the addition of DTP3 to MKK7_KD generates the expected fluorescence quenching effect when the protein was solubilised in phosphate buffer (see Supplementary material Fig. S2D), while the fluorescence of the shorter MKK7_KD domain lacking the N-terminal amino acid region, 101–136, which contains the tryptophan residue 135, was only marginally affected by DTP3 presence.

## 4 Discussion

The complex between GADD45β and MKK7 has been recently developed into a novel therapeutic target in MM. Multiple lines of evidence demonstrate that the small acidic protein, GADD45β, can physically interact with and inhibit the kinase activity of MKK7, an upstream activator of pro-apoptotic JNK kinases. Constitutive GADD45β expression depends upon NF-κB activation and is largely restricted to certain types of cancer cells, such as malignant MM plasma cells. Our recent studies demonstrated that elevated GADD45β expression promotes cancer-cell survival in MM cells, and that disrupting the GADD45β/MKK7 complex spontaneously restores apoptosis by inducing sustained JNK activation.

DTP3, a D-tripeptide isolated through the screening of a library of synthetic tetrapeptides and by a step of chemical optimization, is an effective inhibitor of the GADD45β/MKK7 complex by virtue of its ability to bind to MKK7 with high affinity, resulting in a conformational change that likely causes the displacement of GADD45β [2]. Importantly, DTP3 does not interfere with the MKK7 enzymatic activity [2], thereby enabling it to restore the MKK7 catalytic function, upon displacement of GADD45β, in tissues where GADD45β is highly expressed. Since GADD45β upregulation is dispensable for many of the essential physiological functions of NF-κB in tissue homeostasis, immunity and inflammation, unlike global NF-κB blockade, systemic GADD45β inhibition is generally well tolerated *in vivo*. Due to these properties of its therapeutic target, GADD45β/MKK7, DTP3 displays potent and cancer-selective activity against MM cells, both *in vitro* and *in vivo*, and is not toxic to normal tissues. However, given its peptide-based structure, a potential drawback of DTP3 is its limited bioavailability upon administration by oral route.

Despite previous studies demonstrating the high affinity and target specificity of DTP3 for MKK7, the precise molecular details underlying the DTP3 interaction with MKK7 remain to be determined. The precise characterization of the DTP3/MKK7 interaction is further complicated by the lack of structural information on the GADD45β/MKK7 complex, although molecular models have been previously reported [2,4]. Moreover, it has been previously demonstrated that an intrinsic fluorescence quenching effect occurs upon binding of DTP3 to MKK7, suggesting that DTP3 either directly interacts with the indole ring of a tryptophan residue or the conformational re-arrangement it induces exposes such a residue to fluorescence absorbing centers. Interestingly, this fluorescence quenching effect was observed with both the full-length protein [2] and the kinase domain of MKK7, consistent with the presence of several tryptophan residues in this domain (*i.e.*, residues 101–405, containing W135, W306, and W393).

**Fig. 5:**
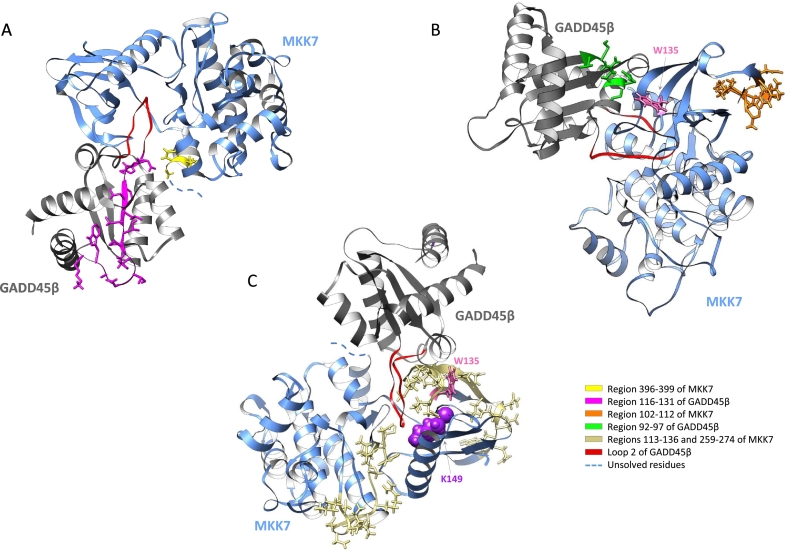
Ribbon representation of the GADD45β/MKK7 complex in which GADD45β is depicted in grey and MKK7 in blue. (A) Regions 396–399 of MKK7 (yellow) (MKK residues from 400 to 405 cannot be represented because their coordinates have not been solved) and 116–131 of GADD45β (magenta) are represented in stick; (B) residues 102–112 of MKK7 are coloured in orange while residues 92–97 of GADD45β are reported in green. In (C) the MKK7 regions (113–136 and 259–274) cross-linked by the photoactivatable SDA-DTP3 are reported in gold. In all figures the loop 2 of GADD45β is reported in red. In (B) and (C), W135 is highlighted in pink and atoms of K149, responsible of ATP binding, are reported as spheres.

Here we investigated the mechanisms underlying the interaction between the kinase domain of MKK7 and GADD45β by using an experimental approach based on MS-based foot-printing and cross-linking mass spectrometry, with the aim to compare the results from MS analyses with the models previously reported for this interaction [2,4]. In addition, we sought to clarify the mechanism by which DTP3 disrupts the GADD45β/MKK7 and identify the DTP3 binding sites on MKK7. The GADD45β/MKK7 interaction has been largely investigated by the use of mutagenesis experiments [4,5] in parallel with molecular modelling studies. Previous studies reported that the interface of the GADD45β/MKK7 complex involved interactions between the so-called acidic loop 1 of GADD45β (residues 61–68) and the basic residues in the MKK7 catalytic pocket. However, based on crystallographic and NMR data available for the kinase domain of MKK7 [[7], [8], [9]] as well as for the GADD45β homologues, GADD45α and GADD45γ [10,11], a more recent molecular model was proposed [2]. This study still confirms that the interaction of GADD45β occurs at MKK7 catalytic pocket but suggests that it is likely mediated by the GADD45β acidic loop 2 (residues 103–117) in place of loop 1. Our data provide the first experimental evidence in support of the model proposed in Tornatore et al. [2], as they indicate insertion of the loop 2 of GADD45β into the enzymatic pocket of MKK7, interaction with the catalytic residues and preclusion of the access of ATP. We found that the peptide 116–131 of GADD45β (Fig. 5A, magenta region), which is contiguous to and partly overlaps with loop 2 (Fig. 4A, red region), is one of two peptides cross-linked to MKK7 upon photo-activation. Interestingly, cross-linking of this peptide occurs with the spatially proximal and external amino acidic region 396–405 of MKK7_KD (Fig. 4A, yellow region), suggesting that loop 2 occupies most of the MKK7 catalytic cavity and that the cross-linking largely occurs on its outer surface, as predicted by previous models. Similarly, the other pair of cross-linked peptides, namely peptide 92–97 of GADD45β (Fig. 4B, green region) and peptide 102–112 of MKK7_KD (Fig. 4B, orange region) are external to the MKK7 catalytic pocket. The relevance of these interactions is further validated by the observation that the two pairs of cross-linked peptides disappeared or were largely reduced in the presence of the GADD45β/MKK7 inhibitor, DTP3. These observations further indicate that these two peptide regions of MKK7 are part of the interaction surface with the tripeptide or, alternatively, are involved in the resulting conformational re-arrangement of the kinase. They are also in keeping with the ability of DTP3 to dissociate the GADD45β/MKK7 complex upon binding to MKK7. The presence of a tryptophan residue (*i.e.*, W135, Fig. 5B, in pink) near the DTP3-interaction region at the N-terminus of the kinase domain is supportive of a model whereby this residue is largely responsible for the quenching effect observed in the presence of DTP3.

Collectively, our results from the experiments of photo-cross-linking of DTP3 and MKK7_KD indicate that the MKK7 N-terminal residues, 113–136, which are contiguous to fragment 102–112, are directly involved in the binding to DTP3 (Fig. 5C, gold regions). These results also suggest that the neighbouring tryptophan residue 135 is largely responsible for the fluorescence quenching effect of DTP3, consistent with the loss of this effect in the N-terminally truncated MKK7_KD protein lacking residues 101–136. A second interaction site of DTP3 with MKK7_KD was identified within the MKK7 region 259–274 which also forms part of the MKK7 interaction surface with GADD45β. The two MKK7 peptides that interact with DTP3 are highlighted in Fig. 5C, together with the catalytic K149 residue responsible for ATP binding (reported as spheres). These data are consistent with a mechanism whereby DTP3 interacts with two spatially adjacent outer MKK7 regions, which together form a shallow pocket that is proximally located to the pocket hosting ATP. In the presence of GADD45β, the DTP3-binding region is partly occupied by the loop 2 of GADD45β, suggesting that the interactions of GADD45β and DTP3 with MKK7 are mutually exclusive.

## 5 Conclusion

Here, we have validated and extended the structural understanding of the interaction between GADD45β and MKK7 and identified the amino acidic regions of MKK7 that bind to DTP3, thereby providing important new insights into the mechanism by which DTP3 disrupts the GADD45β/MKK7 complex. These results, delineating the DTP3-binding pocket of the kinase domain of MKK7, clarify not only how DTP3 inhibits GADD45β/MKK7, but also the basis for the intrinsic fluorescence quenching effect observed in the presence of DTP3 and how the binding of DTP3 does not interfere with the enzymatic activity of MKK7. The further structural characterization of this pocket and the molecular determinants underlying the MKK7 interaction with DTP3 has profound implications not only for the development of novel, second-in-class DTP3-like therapeutics with improved oral bioavailability and potentially other pharmacokinetic and ADME properties, but also for informing potential mechanisms of drug resistance, which might arise in patients treated with DTP3.

## Appendix A Supplementary data

Supplementary material Image 1
