# Supplementary material for: Probing the interaction interface of the GADD45β/MKK7 and MKK7/DTP3 complexes by chemical cross-linking mass spectrometry
Source: Int J Biol Macromol. 2018 Jul 15;114:114–23. doi: 10.1016/j.ijbiomac.2018.03.090 (PMC5981002; doi:10.1016/j.ijbiomac.2018.03.090)
Supplement: Supplementary file 1 — Supplementary material [file mmc1.doc]

**SUPPORTING INFORMATION**

**Probing the interaction interface of the GADD45β/MKK7 and MKK7/DTP3 complexes by chemical cross-linking mass spectrometry**

Camilla Rega^a^, Rosita Russo^a^, Annalia Focà^b^, Annamaria Sandomenico^b^, Emanuela Iaccarino^b^, Domenico Raimondo^c^, Edoardo Milanetti^d^, Laura Tornatore^e^, Guido Franzoso^e^, Paolo Vincenzo Pedone^a^, Menotti Ruvo^b*^ and Angela Chambery^a*^

^a^ Department of Environmental, Biological and Pharmaceutical Sciences and Technologies, University of Campania “Luigi Vanvitelli”, 81100 Caserta, Italy

^b^ CNR-IBB, 80134 Napoli, Italy

^c^ Department of Molecular Medicine, Sapienza University of Rome, 00161, Roma, Italy

^d^ Department of Physics, Sapienza University of Rome, 00161, Rome, Italy

^e^ Department of Medicine, Centre for Cell Signalling and Inflammation, Imperial College London, London W12 0NN, UK

**Table S1.** Peptides identified by UPLC-MS analysis of GADD45β (A) and MKK7_KD (B) tryptic digest. Peptide amino acid sequences and positions, retention times, theoretical and experimental masses together with charge states and masses of the precursor ions are reported.

**Table S2.** Peak integration data reporting normalized areas of tryptic peptides of isolated GADD45β (A) and MKK7_KD (B) compared to those obtained upon GADD45β/MKK7_KD complex formation. Tryptic peptides protected from enzymatic hydrolysis and showing a significant peak intensity decrease upon complex formation are highlighted in grey.

**Table S3.** Peak integration data reporting normalized areas of tryptic peptides of isolated MKK7_KD (M) and in complex with GADD45β (Complex) in the absence (C-M) and in the presence (C1-M1) of DTP3.

**Figure S1.** (A) 12% SDS-PAGE gel analysis of the GADD45β (lane 1) and MKK7_KD (lane 2) recombinant proteins; M, molecular weight markers. MALDI-TOF spectra of GADD45β (B) and MKK7_KD (C) recombinant proteins.

**Figure S2.** (A) CD spectrum of recombinant MKK7_KD at 5.0 μM in 10 mM phosphate buffer pH 7.0. Fluorescence emission spectra of recombinant MKK7_KD at 1.25 μM in 10 mM phosphate buffer pH 7.0 in the presence of increasing concentrations of (B) DTP3 and (C) scrambled peptide (SCRMB). (D) Binding saturation curve obtained by plotting the fluorescence emission values at 333 nm against DTP3 concentration.

**Figure S3.** (A) Biacore sensorgrams obtained by injection of soluble MKK7_KD over immobilized GADD45β. The protein was analyzed at 6, 12, 25, 50, 75 and 100 nM. The dissociation constant was estimated by the corresponding kinetic constants, Kon and Koff, determined by fitting of the sensorgrams data points. (B) Saturation curve related to the binding of MKK7_KD to biotin-GADD45β obtained by ELISA.

**Figure S4.** Base peak intensity chromatogram obtained from the UPLC-MS analysis of GADD45β tryptic digestion. Ion peaks mapping on GADD45β sequence are reported.

**Figure S5.** Base peak intensity chromatogram obtained from the UPLC-MS analysis of MKK7_KD tryptic digestion. Ion peaks mapping on MKK7_KD sequence are reported.

**Figure S6.** Base peak intensity chromatograms obtained from the UPLC-MS analysis of MKK7_KD (A), GADD45β (B) and GADD45β/MKK7_KD complex (C) tryptic digestions. Elution times of peptides protected from enzymatic hydrolysis are indicated by arrows.

**Figure S7.** The predicted model of the interaction between GADD45β (grey) and MKK7_KD (blue) in complex in ribbon representation. The tryptic peptides probed by MS-based foot-printing are localized at interface between GADD45β and MKK7_KD, shown in pink. In yellow are indicated the protected cleavage sites.

**Figure S8.** MS/MS spectrum of the quadruply charged ion at *m/z* 784.384 corresponding to the intermolecular cross-links occurring between the peptide 396-405 of MKK7_KD and the peptide 116-131 of GADD45β.

**Figure S9.** MS/MS spectrum of the quadruply charged ion at *m/z* 656.699 corresponding to the intermolecular cross-links occurring between the peptide 92-97 of GADD45β and the peptide 102-112 of MKK7_KD.

**Figure S10.** (A) Analytical RP-HPLC analyses performed at different incubation times for monitoring the NHS-reaction of DTP3 with SDA. (B) Preparative RP-HPLC for the purification and (C) mass spectrometry characterization of the SDA-DTP3 product.
